# Medical and Philosophical Intelligence

**Published:** 1814-02

**Authors:** 


### MEDICAL AND PHILOSOPHICAL INTELLIGENCE.

*ROYAL SOCIETY.*—At the first sitting of the Royal Society this season, on Thursday the 4th of November, a description of a sliding scale of chemical equivalents, contrived by Dr. Wollaston, was read.

It was first observed by Richter, that when two neutral salts are made to decompose each other, the neutrality of neither is disturbed. Thus, if you dissolve 100 grains of sulphate of potash in water, and pour into the solution muriate of barytes in sufficient quantity to decompose the whole of the sulphate, two new neutral salts will be formed, namely, sulphate of barytes and muriate of potash. If into the muriate of potash thus formed, a sufficient quantity of nitrate of silver be dropped to decompose it, two neutral salts will be formed, namely, nitrate of potash and muriate of silver. Thus the same weight of potash has been united in succession with sulphuric, muriatic, and nitric acids. The weight of barytes that neutralized the muriatic acid neutralized likewise the weight of sulphuric acid combined with the potash which had neutralized the sulphuric acid. These observations of Richter were still farther generalized by Berthollet; but it is to Dalton that we owe the full generalization of the facts, aud the explanation of them. He supposes that bodies unite atom to atom, and showed how the weights of the atoms of bodies might be determined.

Dr. Wollaston has divided the slider of a scale into the logarithmic spaces from 10 to 320, by a method familiar to all who are acquainted with the nature of logarithms, or in a similar manner as the line of numbers is laid down in Gunter's scale. He considers 10 as representing the weight of an atom of oxygen. On both sides of the scale he has written the substances most familiar to chemists, viz. the acids, bases, water, and principal salts, each opposite to the number on the slide, which corresponds with the weight of an atom of it. Suppose we want to know how much oxygen combines with 200 mercury: bring 200 on the slide opposite to *mercury* on the scale; then over against *oxygen* on the slide will be found 16, the quantity of of oxygen required. Suppose we want to know how much oxide of copper combines with 60 of sulphuric acid: bring sulphuric acid opposite to 60, and we shall find over against oxide of copper 61, the number required. The sliding rule is fitted to answer an infinite number of similar questions.

Dr. Wollaston determined experimentally the composition of nitrate of potash, and found it composed of 68 acid + 59 potash. His scale is referred to carbonate of lime, which he considers as the most exact standard of comparison that can be obtained.

On Thursday, the 11th of November, part of the Croonian Lecture on the *Influence of the Nervous System on Muscular Motion*, by B. C. Brodie, Esq. was read. This lecture began with a short historical account of the facts and opinions of former physiologists on this subject. The author then relates a number of experiments which he himself instituted still farther to advance the subject. He found that after the destruction of the lower part of the spinal marrow of a dog, the arterial blood of a horse injected into the lower extremities of the animal, after circulating through the limb, came out at the mouths of the veins dark colored. He found that the lower extremities of a frog, treated in the same way, though deprived of voluntary motion, contracted by stimuli and by the galvanic influence. He found that the heart of a rabbit continued to beat with its usual regularity for some time after the blood-vessels had been emptied of their contents; but that the action of the heart was destroyed when an animal was strangled, showing clearly that the stimulus of the blood is not the exciting cause of the action of the heart.

On Thursday, the 18th of November, the remainder of Mr. Brodie's paper on the *Influence of the Nervous System on Muscular Motion, particularly of the Heart*, was read. The author concluded with some general inferences from the experiments detailed in the preceding part of his paper.

---

*Account of the New Rupture Society, for the Relief of both Sexes afflicted with Herniary Complaints and Prolapses.*—This Society was instituted on the 15th of May, 1804, by a respectable meeting of benevolent gentlemen, convened at the Freemasons' Tavern; and patients were admitted on the 6th of July, 1805, since which time the number of persons applying for trusses has been rapidly and progressively increasing.

The *yearly* number of patients hitherto attended by the surgeon of this Charity, most of whom were totally destitute of the necessary apparatus for their personal safety, is as follows:

| | | | | | |
|---|---|---|---|---|---|
| From July the 6th, | 1805 | to | 1806 | No. | 76 |
| | 1806 | to | 1807 | — | 252 |
| | 1807 | to | 1808 | — | 407 |
| | 1808 | to | 1809 | — | 399 |
| | 1809 | to | 1810 | — | 498 |
| | 1810 | to | 1811 | — | 554 |
| | 1811 | to | 1812 | — | 669 |
| | 1812 | to | 1813 | — | 903 |

The

The following comparative statement, taken from an exact register of *four thousand two hundred and forty-two* patients relieved by the New Rupture Society, to January 1, 1814, will show the proportion and species of herniary cases occurring in each sex:

| | | Males. | Females. |
|---|---|---|---|
| Ruptures | In both groins | 946 | 19 |
| | In both thighs | 4 | 39 |
| Inguinal | On the right side | 1563 | 51 |
| | On the left side | 927 | 34 |
| Femoral | On the right side | 19 | 139 |
| | On the left side | 11 | 93 |
| | Navel ruptures | 32 | 177 |
| | Ventral herniæ | 3 | 13 |
| | Intestinal prolapses | 22 | 21 |
| | Uterine prolapses | 0 | 128 |
| | Prolapse of the urinary bladder | 0 | 1 |
| | | 3527 | 715 |
| | | | 3527 |
| | Total number of cases | | 4242 |

It is difficult to ascertain at what age persons are most liable to become ruptured; probably, however, during the middle period of life: but, not fewer than one hundred and seventy of the above patients (nearly all males) were ruptured prior to their birth, about the proportion of a fifth of whom had double inguinal herniæ, and one tenth had navel ruptures. Happily, these congenital cases admit of perfect cure, by the application of a compress during the state of infancy.

Of the females afflicted with prolapses of the bowel, sixteen had also uterine prolapses, seven were ruptured, and one had a prolapse of the urinary bladder. Many of the patients having ruptures in the navel, had at the same time inguinal or femoral herniæ; and two women had also prolapsus ani. A person who labored under a ventral rupture, had likewise two inguinal herniæ. Several females, having prolapses of the uterus, had also herniæ on one or both sides. Five men had prolapses, with inguinal ruptures. Another male subject had one inguinal and two femoral ruptures, produced by sudden bodily exertion: and, in many men, hydrocele was combined with their ruptures; all which accessory cases were cured, or relieved, by appropriate surgical operations.

By an order of the Governors, notice has been publicly given to parochial officers, superintendents of hospitals, and the conductors of other charitable institutions, that the benefits of this Society are extended to all parts of the kingdom, provided exact descriptions of the cases, with proper measurements of the patients, be sent by any subscriber, post paid, to Mr. Blair, Surgeon, No. 69, Great Russel-street, Bloomsbury-square. In consequence of this order, the heads of several parishes and public institutions have subscribed to the

New Rupture Society, and thus are enabled to relieve their own afflicted poor at a small expense.

*Royal College of Surgeons.*—The annual course of Lectures on Human and Comparative Anatomy, and Surgery, commenced on Tuesday the 25th of January, the first lecture being given by Mr. Abernethy. The lecturer began by paying a neat and well-merited compliment to the talents of his predecessor in the chair, and his early preceptor, Sir William Blizard, whose maxims concerning the search of truth he strongly inculcated on his hearers. He then explained the distinction between theory and hypothesis, and shewed how absurd it is to rail against the former, from its occasional misapplication and perversion, since theory consists in the inferences which are drawn from a connected series of facts, and is, in truth, nothing more or less than thinking; and it was by such thinking, Mr. Abernethy observed, that Sir Isaac Newton was enabled, from the falling of an apple, to deduce and establish the great law of gravitation. This led him, by a natural transition, to review the claims of Mr. Hunter to the character of a thinking man, and of a man of genius. These he warmly vindicated, declaring his conviction that Mr. Hunter had trodden in the right path of research, and avowing his intention to follow Mr. Hunter's system, because he believed it to be the true one. He now proceeded to consider the structure of organized bodies, beginning with the simple fibre, in which the principle of all their actions resides. After stating the opinions and observations of Haller and others, respecting the form and continuity of this fibre, as it is seen in muscles, to which he attached but little importance, he briefly discussed the various conjectures which had been formed with regard its contractile power. Finding that in all animated bodies this power belongs to the fibres, however it may vary in its composition, he could not admit it to be a mere property of animal matter, but he conceived that it depended on some subtle agent, like electricity, or magnetism, which pervaded these bodies, and endowed them with life and action. Of this hypothesis (for notwithstanding Mr. A.'s application of the term, we will not venture to call it theory) he adduced, towards the conclusion of the lecture, a variety of ingenious illustrations, into which our limits preclude us from following him. The whole lecture was delivered in a very eloquent and impressive manner.

Mr. Bythell, surgeon at Chester, recommends the use of the elastic gum catheter immediately after the operation of lithotomy, which, after considerable experience, he thinks productive of beneficial effects. Wearing the elastic gum catheter assists in conveying the urine direct from the bladder, and allows those parts wounded by the operation an opportunity of healing much quicker than otherwise they would do. It prevents the growth of excrescences and other obstructions in the urethra, which often occur, and are frequently attended with unpleasant consequences. The instrument is readily introduced, and occasions little inconvenience.

Dr. Sherwen has ascertained that every good property of stramonium nium may be expected from a similar use of common white poppy heads, the smoke of which, whether swallowed or inhaled, is equally anodyne, and less deleterious than that of stramonium. To obtain the poppy heads in perfection, they should be carefully dried while green, before they have attained their greatest magnitude. It is probable that the green leaves, well dried, would also be efficacious. Dr. S. also observes, that similar effects would probably result from the dried leaves of digitalis, particularly in that species of asthma connected with œdematous ankles, irregular pulse, and other symptoms of hydrothorax.

---

According to the report of several *Apothecaries* residing in various districts of the metropolis, the following was the average of the diseases in London between November 20th and December 19th:—

| | | | |
|---|---|---|---|
| Anasarca | 13 | Erysipelas | 17 |
| Ascites | 4 | Enteritis | 7 |
| Asthma | 93 | Entrodynia | 1 |
| Acne | 7 | Enuresis | — |
| Apoplexia | 8 | Epilepsia | 4 |
| Aphtha lactantium | 7 | Febris remittens | 48 |
| Amenorrhœa | 19 | ——— intermittens | 5 |
| Asthenia | 26 | ——— puerpera | — |
| Abortio | 2 | Gastritis | 4 |
| Bronchitis acuta | 108 | Gastrodynia | 4 |
| ——— chronica | — | Hæmatemesis | 2 |
| Cancer | 8 | Hæmoptoe | 10 |
| Chlorosis | 16 | Hæmorrhois | 1 |
| Cholera | 10 | Hepatitis | 18 |
| Chorea | 1 | Hysteria | 16 |
| Cephalalgia | 24 | Hysteritis | — |
| Colica | 17 | Hydrophobia | — |
| Catarrhus | 287 | Hydrocephalus | 4 |
| Convulsio | 5 | Hydrothorax | 14 |
| Carditis | — | Herpes | 13 |
| Cynanche Tonsillaris | 42 | Hypochondriasis | 14 |
| ——— maligna | 4 | Icterus | 8 |
| ——— Trachealis | 6 | Impetigo | 1 |
| ——— parotidea | 10 | Ischuria | 1 |
| Diabetes | 1 | Leucorrhœa | — |
| Dysenteria | 13 | Lithiasis | 1 |
| Diarrhœa | 49 | Lepra | 3 |
| Dyspnœa | 38 | Lichen | 2 |
| Dyspepsia | 70 | Mania | — |
| Dysuria | 7 | Menorrhagia | 17 |
| Ecthyma | 7 | Miliaria | — |
| Eczema | 2 | Morbi Infantiles | 73 |
| Erythema nodosum | 1 | ——— Biliosi* | 75 |

* Morbi Biliosi is meant to comprise those complaints that are popularly called *bilious*; but which do not properly come under any class of diseases.

| | | | |
|---|---|---|---|
| Nephritis | 4 | Rheumatismus chronicus | 46 |
| Obstipatio | — | Rubeola | 17 |
| Ophthalmia | 43 | Scabies | 57 |
| Paralysis | 7 | Scarlatina simplex | 1 |
| Phthisis Pulmonalis | 23 | ——— anginosa | 8 |
| Pemphigus | — | ——— maligna | 4 |
| Pertussis | 14 | Splenitis | 2 |
| Peritonitis | 5 | Scrofula | 33 |
| Pompholyx | — | Tabes Mesenterica | 10 |
| Phrenitis | — | Tetanus | 1 |
| Pneumonia | 97 | Trismus | — |
| Podagra | 12 | Typhus | 14 |
| Porrigo larvalis | 6 | Variola | 9 |
| ——— scutulata | 7 | Varicella | 10 |
| ——— favosa | 6 | Vermes | 10 |
| Psoriasis | 9 | Vertigo | 15 |
| Purpura | 1 | Urticaria | 3 |
| Pyrosis | 4 | | |
| Rachitis | 1 | Total | 1792 |
| Rheumatismus acutus | 59 | | |

The Deaths in the Bills of Mortality from November 24th to December 28th, 1813, according to the returns of the *Parish Clerks*, were as under:—

| | |
|---|---|
| Males . . . . | 1271 |
| Females . . . | 1135 |
| Total | 2406 |

The French Gazette of Health for November contains a detailed relation of the sufferings of a young girl of eight years old, into whose ear a spider had crawled. She experienced momentarily a nervous fit resembling epilepsy, of which the strength gradually increased, impressing those around her with a fear that it would speedily become fatal. The surgeon, unable to extract the spider, poured oil of olives into the ear: hereupon she experienced some convulsive movements, longer and more violent than those she had before; and when they terminated, she was quite relieved.

Professor Mangeli has published in the Milan Journal a long report upon the action of the venom of vipers. He states, as the result of his experience, that ammoniac is the only sovereign remedy for the bite of those reptiles, and that opium and musk, which have been hitherto prescribed by Italian physicians, have no certain effect.

An Havannah paper mentions that an aged priest, in Guatimala, had lately applied himself to the production of opium in that province, and had succeeded to a degree that promised to make his discoveries a great national benefit. The Guatimala opium was said to be of a much superior quality to that obtained from the Levant.

Mr.

Mr. W. T. Ward, Secretary to the Committee of Apothecaries, &c. has requested us to announce, that the new Bill is framed by the London Committee, and is now in the hands of counsel to arrange, and give the leading details, preparatory to its being submitted to the medical public.

*Medical and Chemical Lectures and Examinations.*—Dr. Hooper and Dr. Ager will begin their next Course on the Theory and Practice of Physic, Chemistry, and the Materia Medica, at the Theatre, 26, Cork-street, Burlington Gardens, on Monday, February 7th, at eight o'clock in the morning. Three Courses are given every year, beginning in February, June, and October, each on the first Monday of the month.

The Lectures on the Teeth, which had during many years been delivered by Mr. Moor, will be continued by his successor Mr. Fuller, Surgeon-Dentist to H. R. H. the Duchess of York. The Spring Course will commence the latter end of February, at his house, No. 6, Palsgrave-place, Temple Bar.

Dr. Adams will commence his Spring Course of Lectures on the Institutes and Practice of Medicine, on Tuesday the 1st of February, at ten o'clock, at his house, 17, Hatton Garden.

Dr. Prout will commence a Course of Lectures on Animal Chemistry, on Friday, February 18th, at half-past eight in the evening, at his apartments, 4, Arundel-street, Strand; which will be continued weekly at the same hour.

Dr. Gooch will commence his third Course of Lectures on Midwifery, and on the Diseases of Women and Children, early in February, at the Medical Theatre of St. Bartholomew's Hospital.

*Books in the press.*—Dr. Adams's long projected work on the erroneous Opinions and consequent Terrors usually entertained concerning Hereditary Diseases. Connected with the subject, are some remarks on the attempts at reducing cutaneous diseases to classes and orders, and on the unnecessary revival of exploded Greek terms.

An Essay on Medical Economy, comprising a sketch of the state of the profession in England, and the outlines of a plan calculated to give to the medical body in general an increase of usefulness and respectability.

A Practical Essay on the Diseases of the Vessels and Glands of the Absorbent System, being the substance of Observations to which the Prize for 1812 was adjudged by the London College of Surgeons; by William Goodlad, Surgeon, Bury, Lancashire, Member of the College.

The Morbid Anatomy of the Liver, Part 2; by J. R. Farre, M.D.

Mr. C. M. Clarke, Member of the College of Surgeons, shortly will publish Observations on those Diseases of Females that are attended by Discharges.

Mr. Stevenson, Surgeon-Oculist and Aurist to her Royal Highness the Princess of Wales, has in the press, and nearly ready for publication, a greatly enlarged edition of his Treatise on Cataract.

